# S04-4 Tracking participants in the Jackpot.fit program and progress in dissemination of the Austrian sport promotion initiative

**DOI:** 10.1093/eurpub/ckac093.020

**Published:** 2022-08-29

**Authors:** Bernhard Novak, Lena Großschädl, Christian Lackinger, Wolfgang Ruf, Albert Strehn, Sylvia Titze

**Affiliations:** 1 University of Graz, Institute of Human Movement Science, Graz, Austria; 2 Head Office Styria, Social Security Service for Entrepreneurs, Graz, Austria; 3 Kuratorium Wiener Pensionisten-Häuser, Vienna, Austria; 4 Institute of Sport Science, German University of Health and Sport, Berlin, Germany; 5 Head Office Burgenland, Social Security Service for Entrepreneurs, Eisenstadt, Austria

**Keywords:** physical activity, standardized sports club program, adherence, health care sector

## Abstract

**Background:**

In 2015 a co-operation between the social insurance and the organized sport has been established in order to install Jackpot.fit, a standardized sports club based program, initially in 8 regions in Styria, a federal state of Austria. Inactive adults receive information about the long-term exercise program during a 3-week residential stay in a health resort. We aim to investigate who attends Jackpot.fit, the adherence to the program in 2019 as well as the progress of dissemination of Jackpot.fit since 2016.

**Methods:**

Participantś personal data were collected by telephone at the registration. Instructors documented the participants' attendance each single session with a standardized form. The number of participating regions as well as courses within the regions were monitored by the evaluator.

**Results:**

Between 2016 and 2019 2624 people (70.8% female; age: M = 57.1, *SD* = 9.1 years; BMI = 25.8, *SD* = 4.3 kg/m^2^) registered for Jackpot.fit and 84.3% (n = 2212) attended as participants in at least one session. Between September and December 2019, 824 people registered for the courses with an adherence rate as follows: 291 (35.3%) visited 75% or more, 200 (24.3%) between 50% and 74.9% and 232 (28.2%) less than 49.9% of all 638 provided sessions. 101 (12.3%) registered but never attended once. The number of regions rose from 8 in 2016 to 28 in 2019 and the number of courses from 25 to 55 over the same period.

**Conclusions:**

According to the total number of participants, regions, and courses the dissemination moves forward. Further strategies to improve adherence must be developed.

**Acknowledgement:**

The project is supported by the Gesundheitsfonds Steiermark and the Bundessport Förderungsfonds.

